# Supplementary material for: Mutations in VPS18 lead to a neutrophil maturation defect associated with disturbed vesicle homeostasis
Source: Cell Death Dis. 2026 Jan 12;17(1):180. doi: 10.1038/s41419-025-08338-w (PMC12876832; doi:10.1038/s41419-025-08338-w)

# Figure 3D

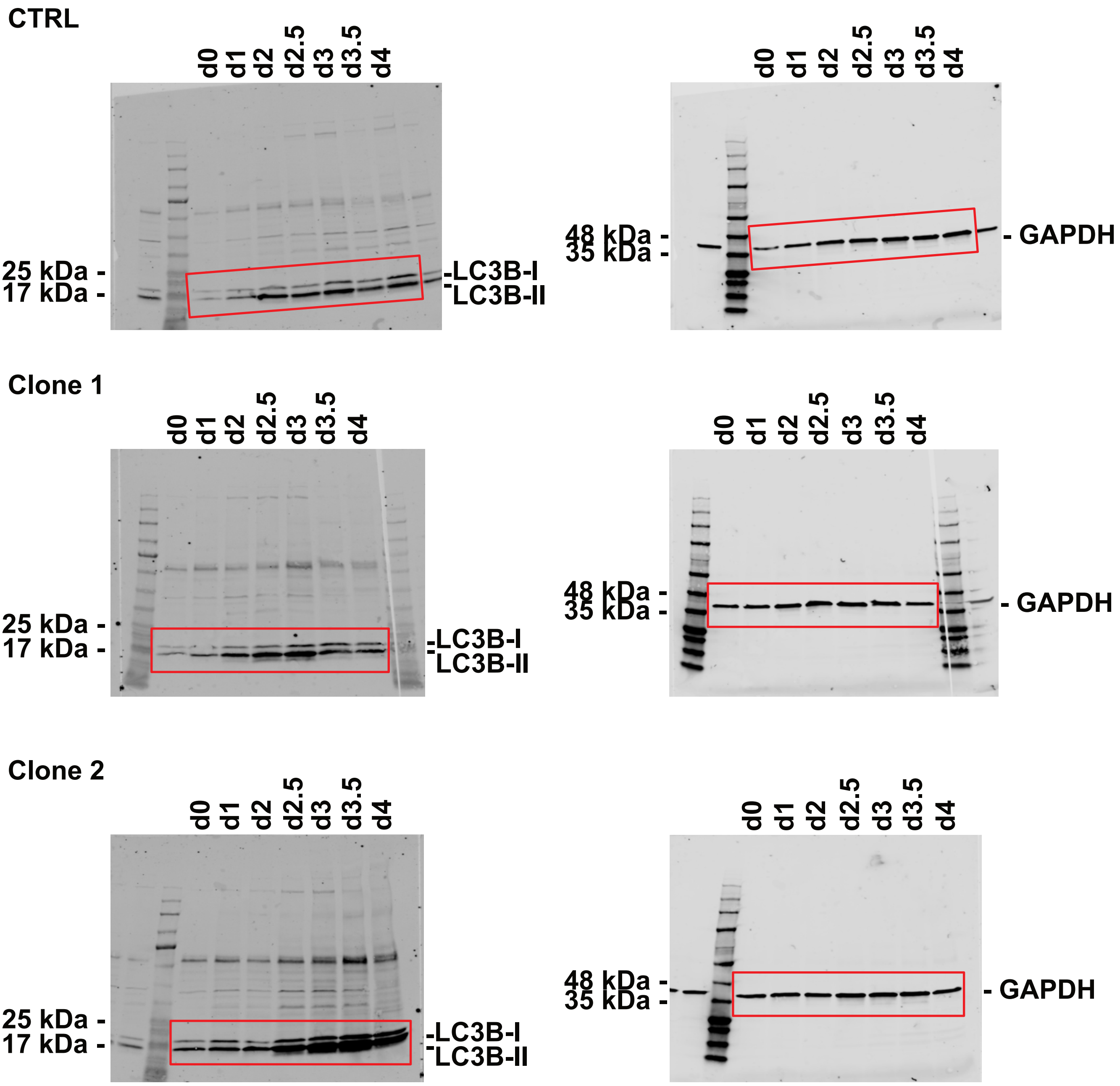

CTRL
d0 d1 d2 d2.5 d3 d3.5 d4
25 kDa -
17 kDa -
-LC3B-I
-LC3B-II
d0 d1 d2 d2.5 d3 d3.5 d4
48 kDa -
35 kDa -
- GAPDH
Clone 1
d0 d1 d2 d2.5 d3 d3.5 d4
25 kDa -
17 kDa -
-LC3B-I
-LC3B-II
d0 d1 d2 d2.5 d3 d3.5 d4
48 kDa -
35 kDa -
- GAPDH
Clone 2
d0 d1 d2 d2.5 d3 d3.5 d4
25 kDa -
17 kDa -
-LC3B-I
-LC3B-II
d0 d1 d2 d2.5 d3 d3.5 d4
48 kDa -
35 kDa -
- GAPDH

## Figure 5C

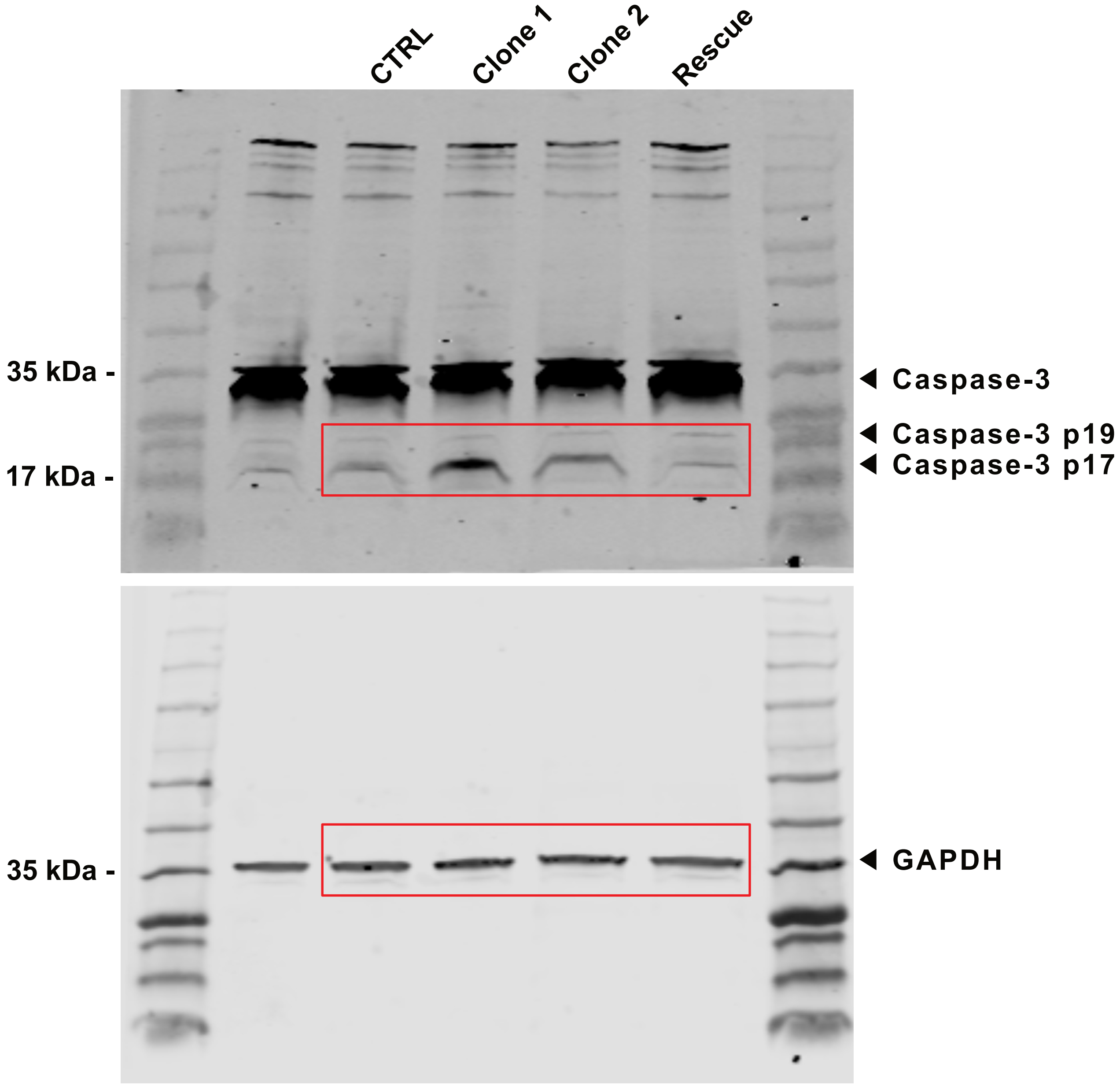

CTRL
Clone 1
Clone 2
Rescue
35 kDa -
17 kDa -
Caspase-3
Caspase-3 p19
Caspase-3 p17
35 kDa -
GAPDH

Figure 6F

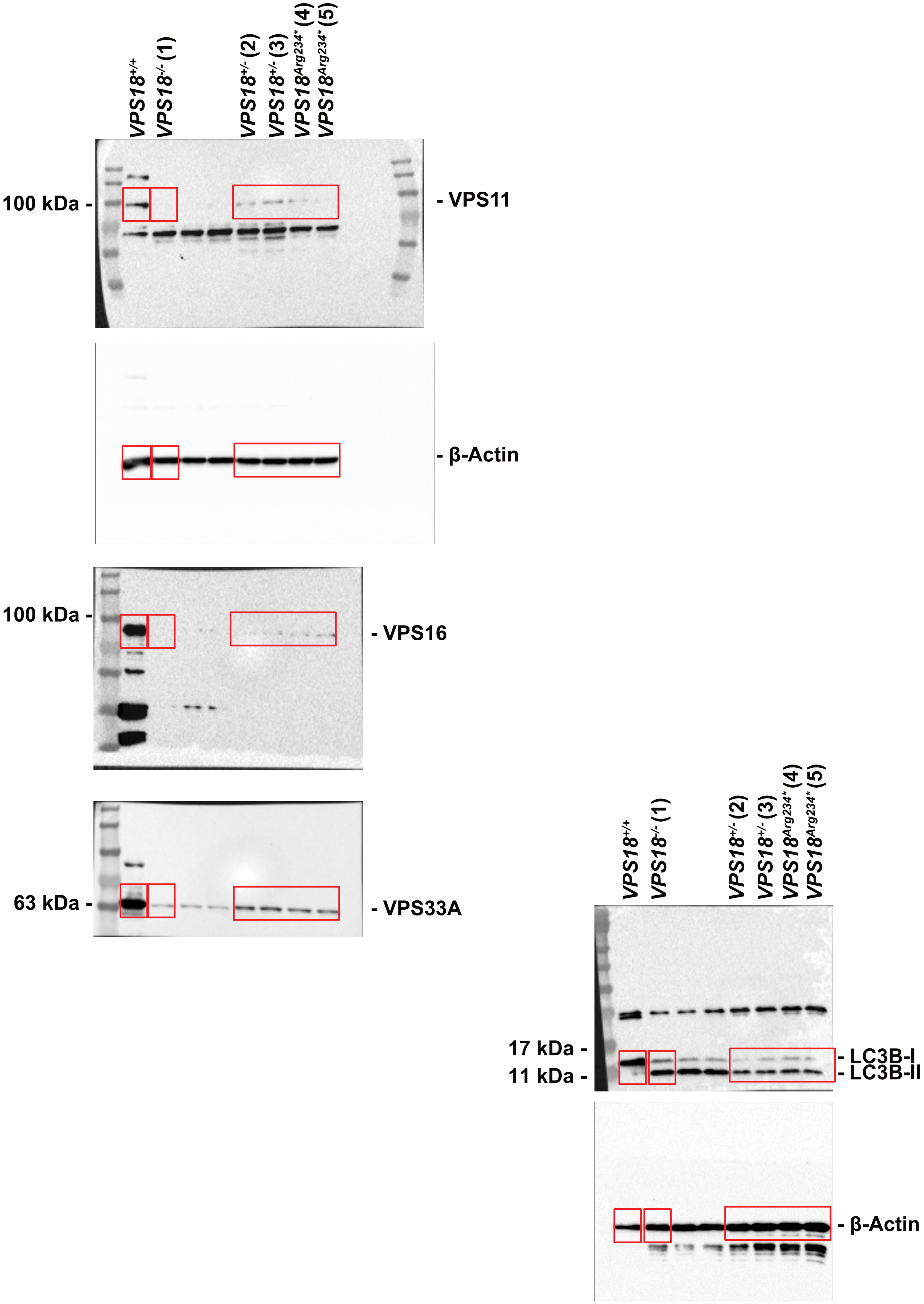

VPS18+/+
VPS18-/- (1)
VPS18+/- (2)
VPS18+/- (3)
VPS18Arg234* (4)
VPS18Arg234* (5)
100 kDa -
- VPS11
- β-Actin
100 kDa -
- VPS16
63 kDa -
- VPS33A
17 kDa -
11 kDa -
- LC3B-I
- LC3B-II
- β-Actin

## Supplementary Figure 1A

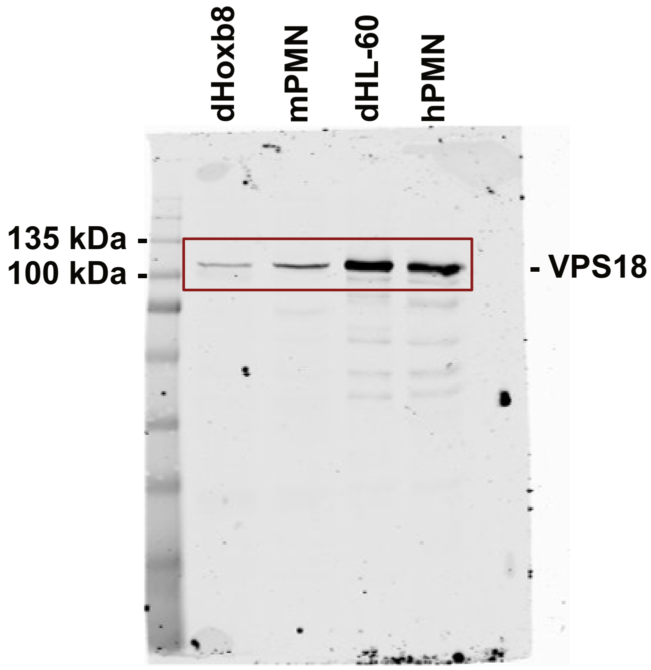


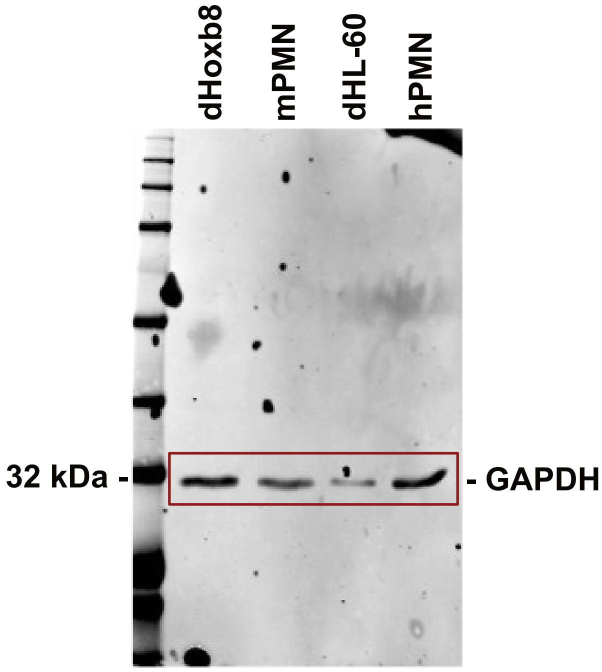


## Supplementary Figure 1E

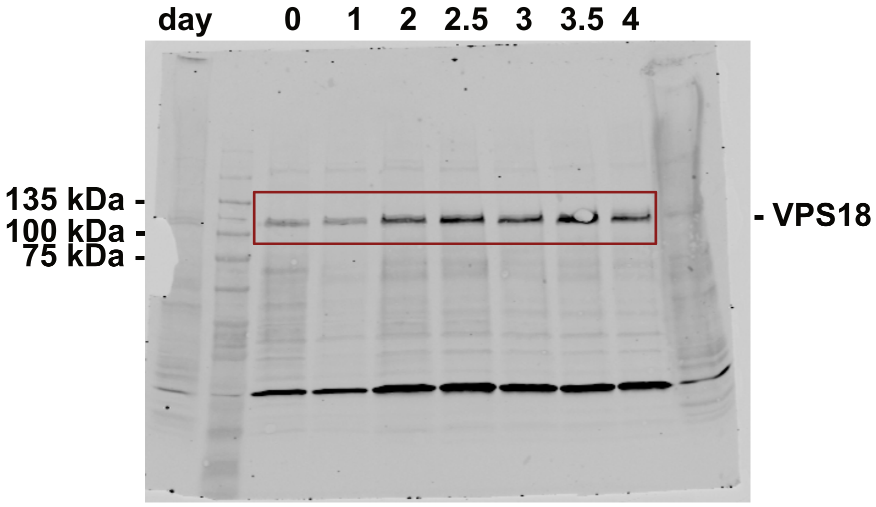


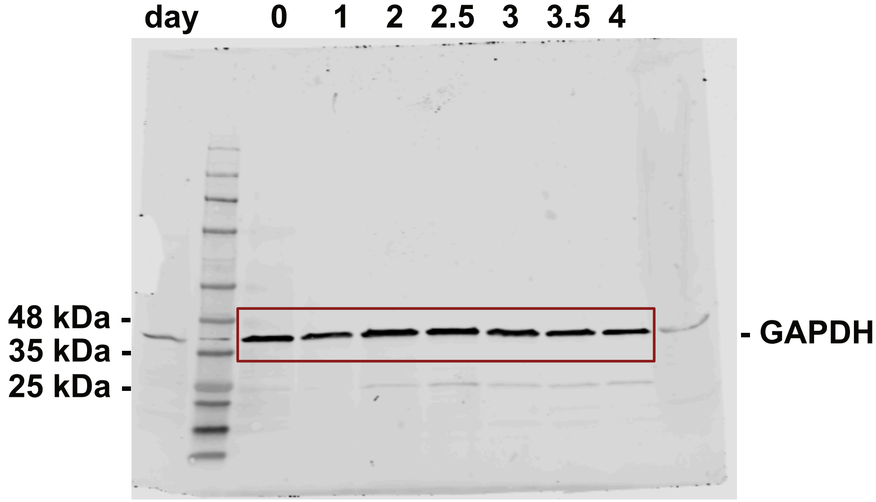

## Supplementary Figure 1G

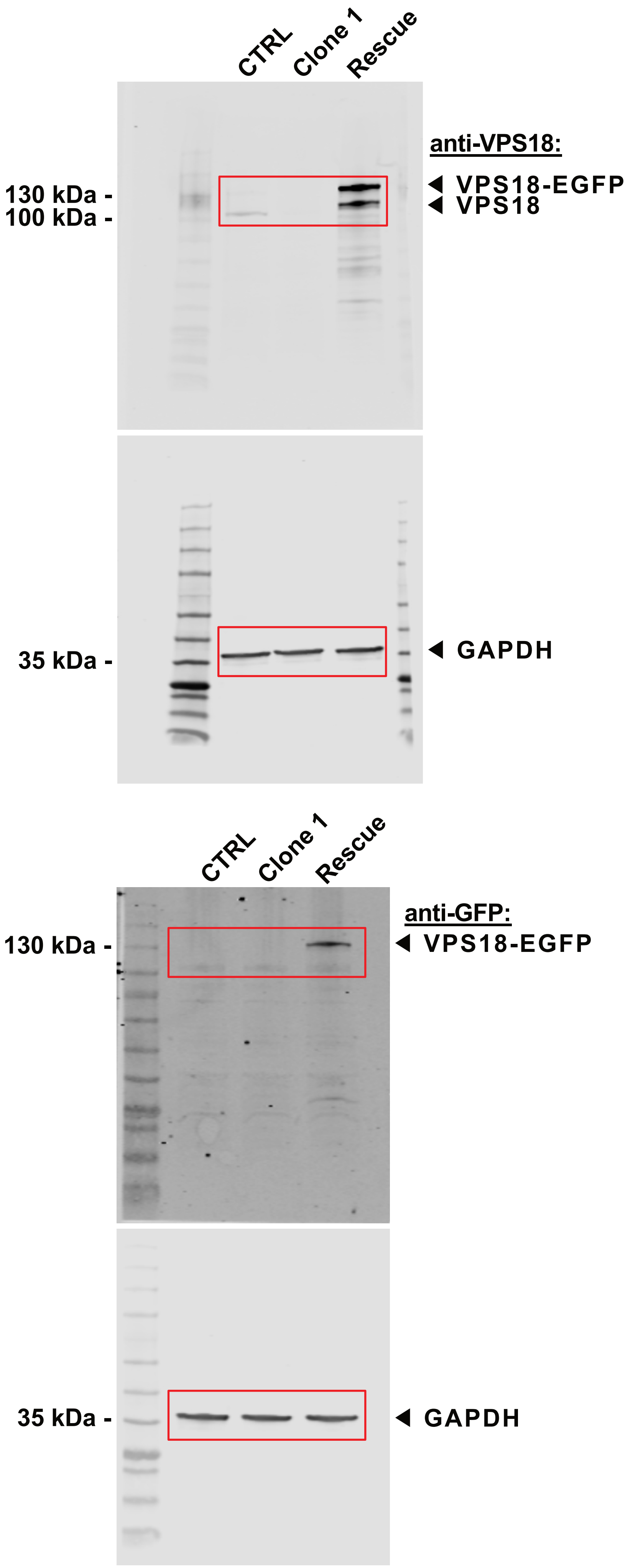

# Supplementary Figure 3C

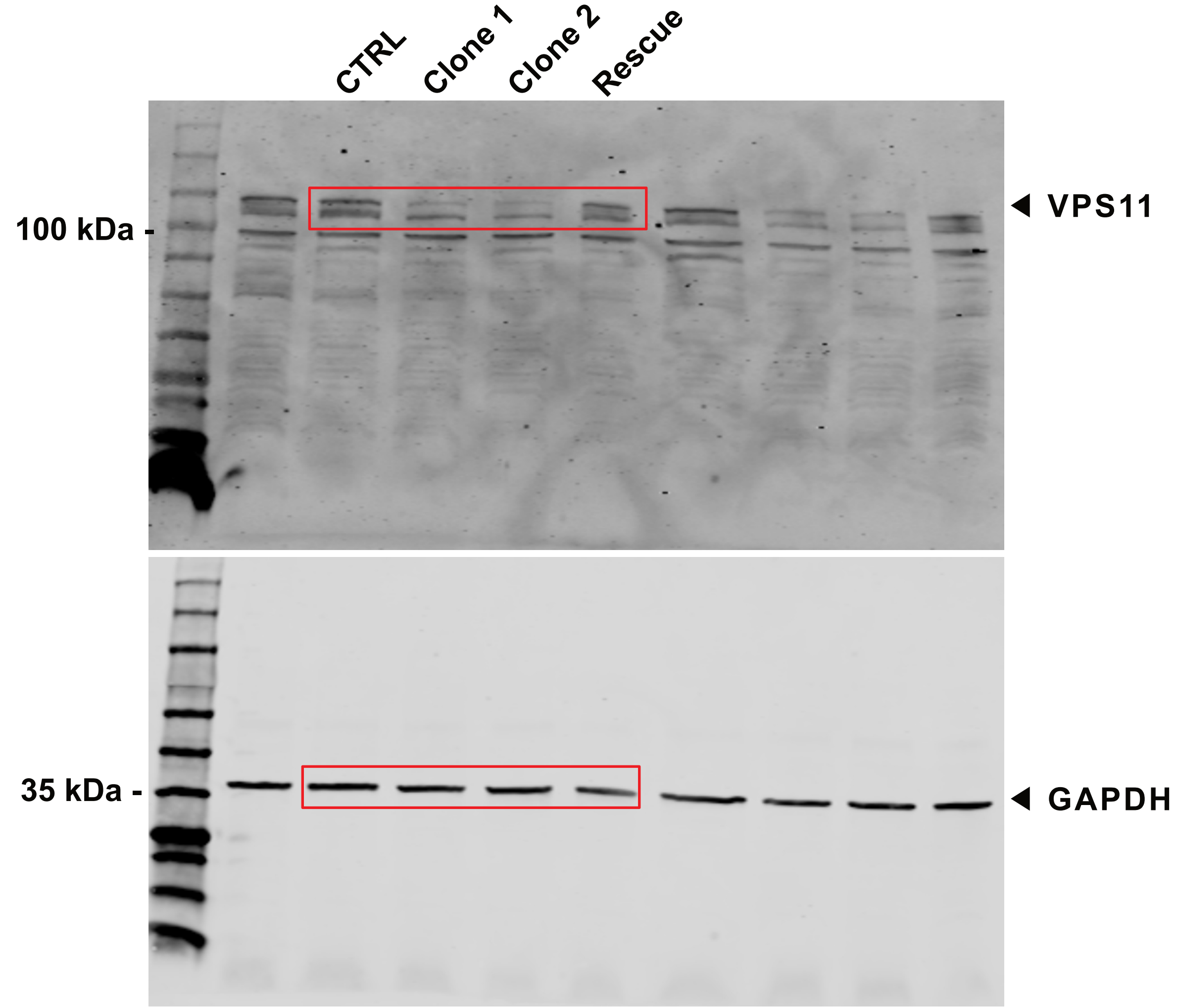


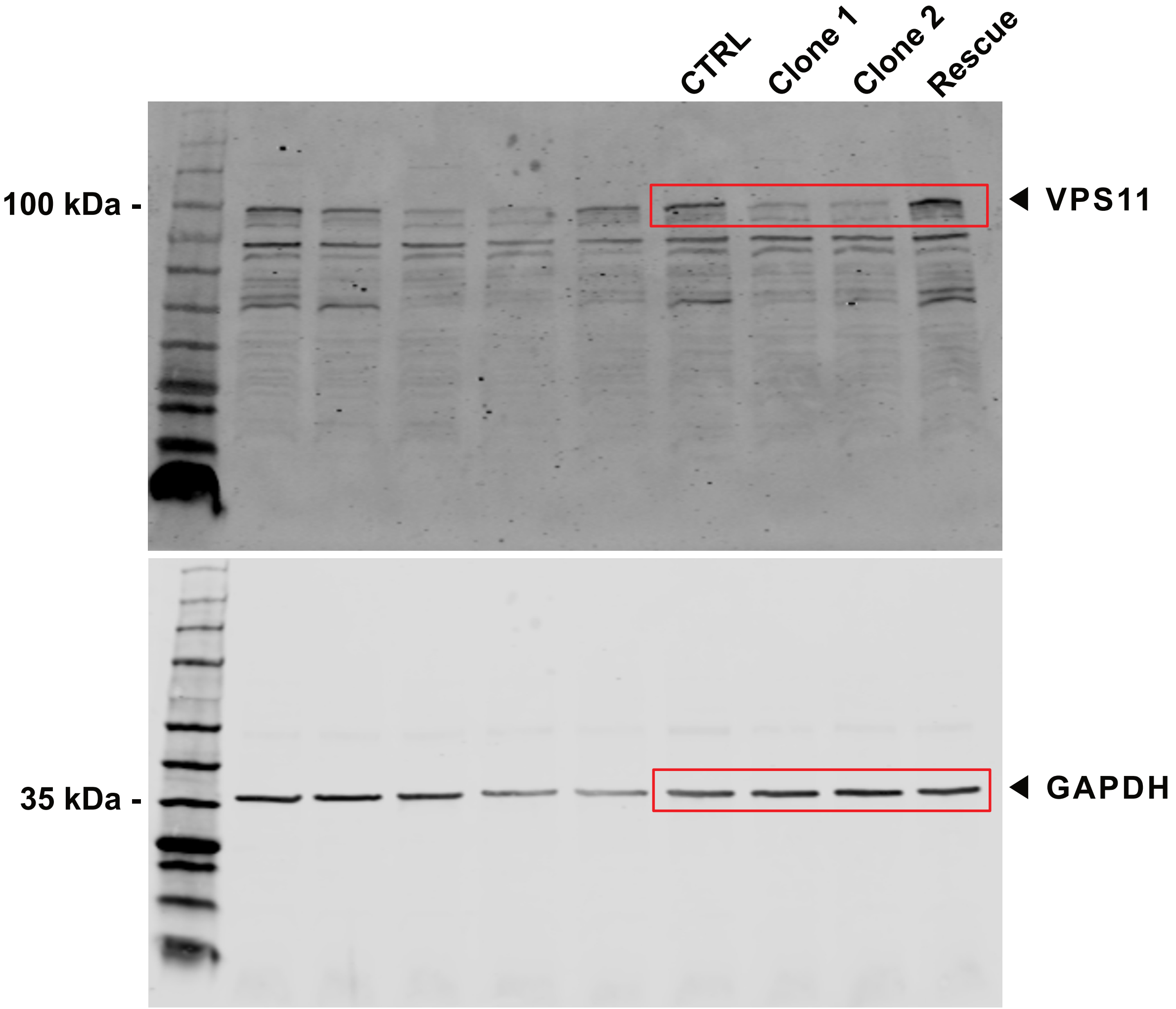

# Supplementary Figure 3C

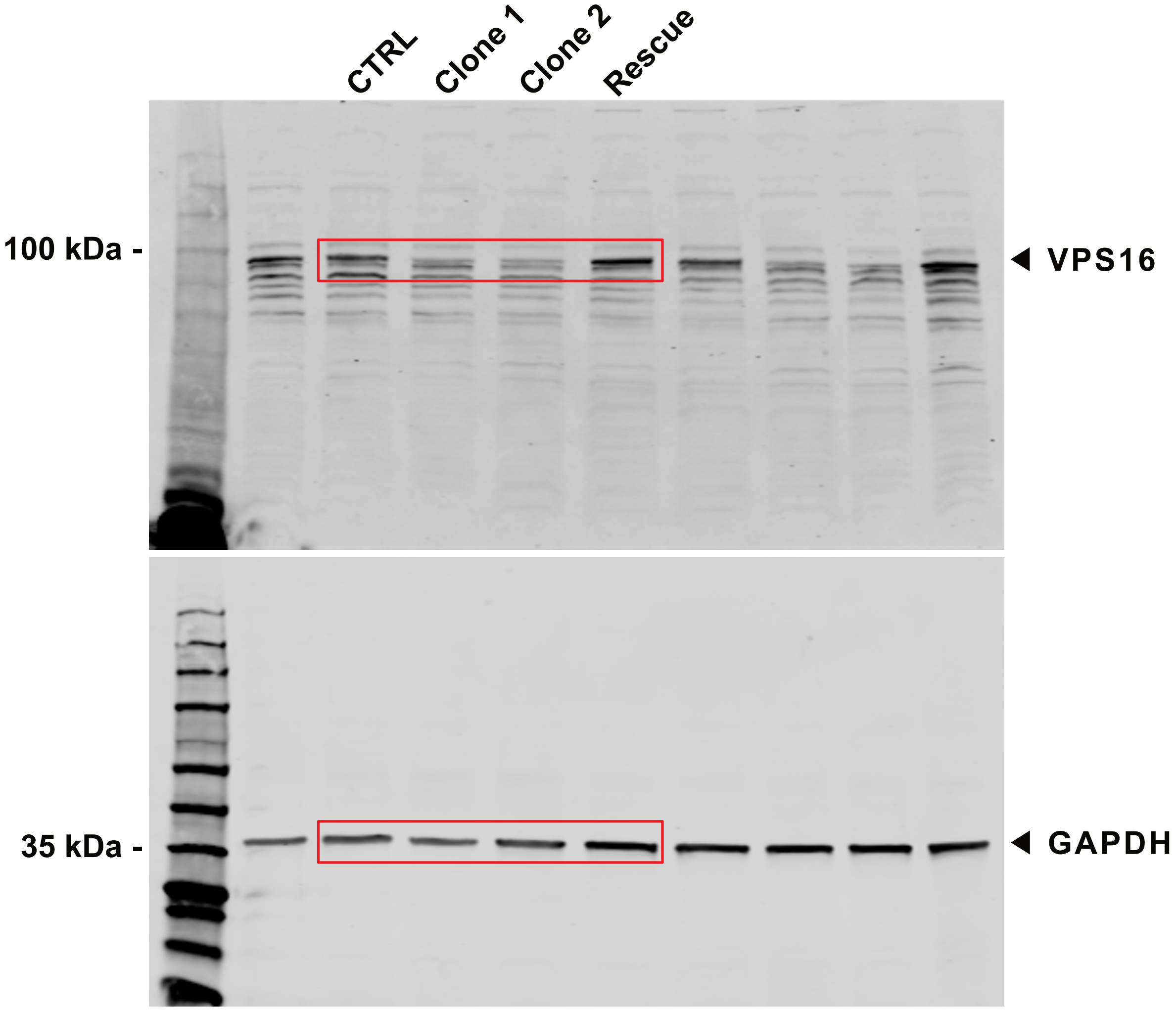


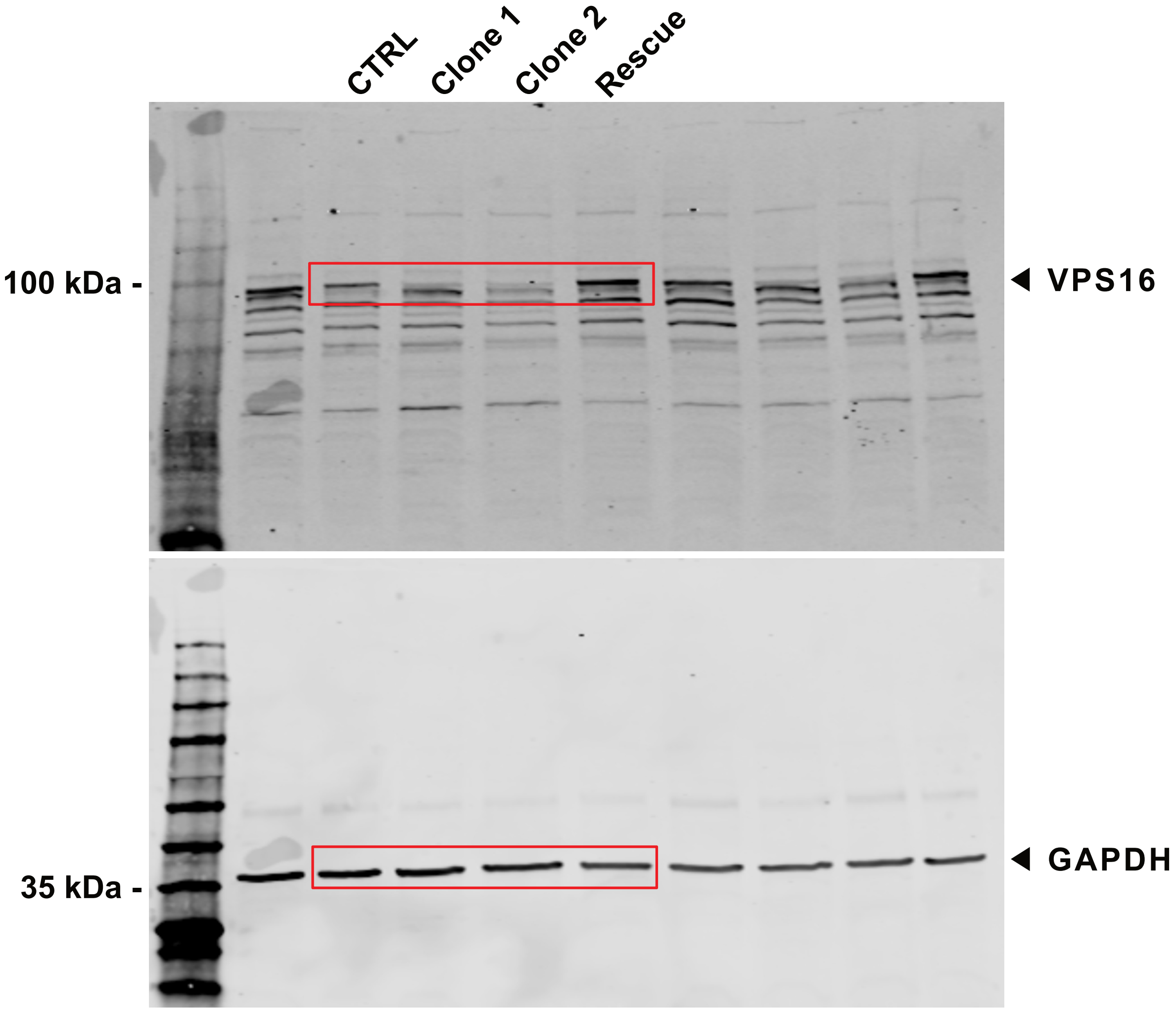

## Supplementary Figure 3C

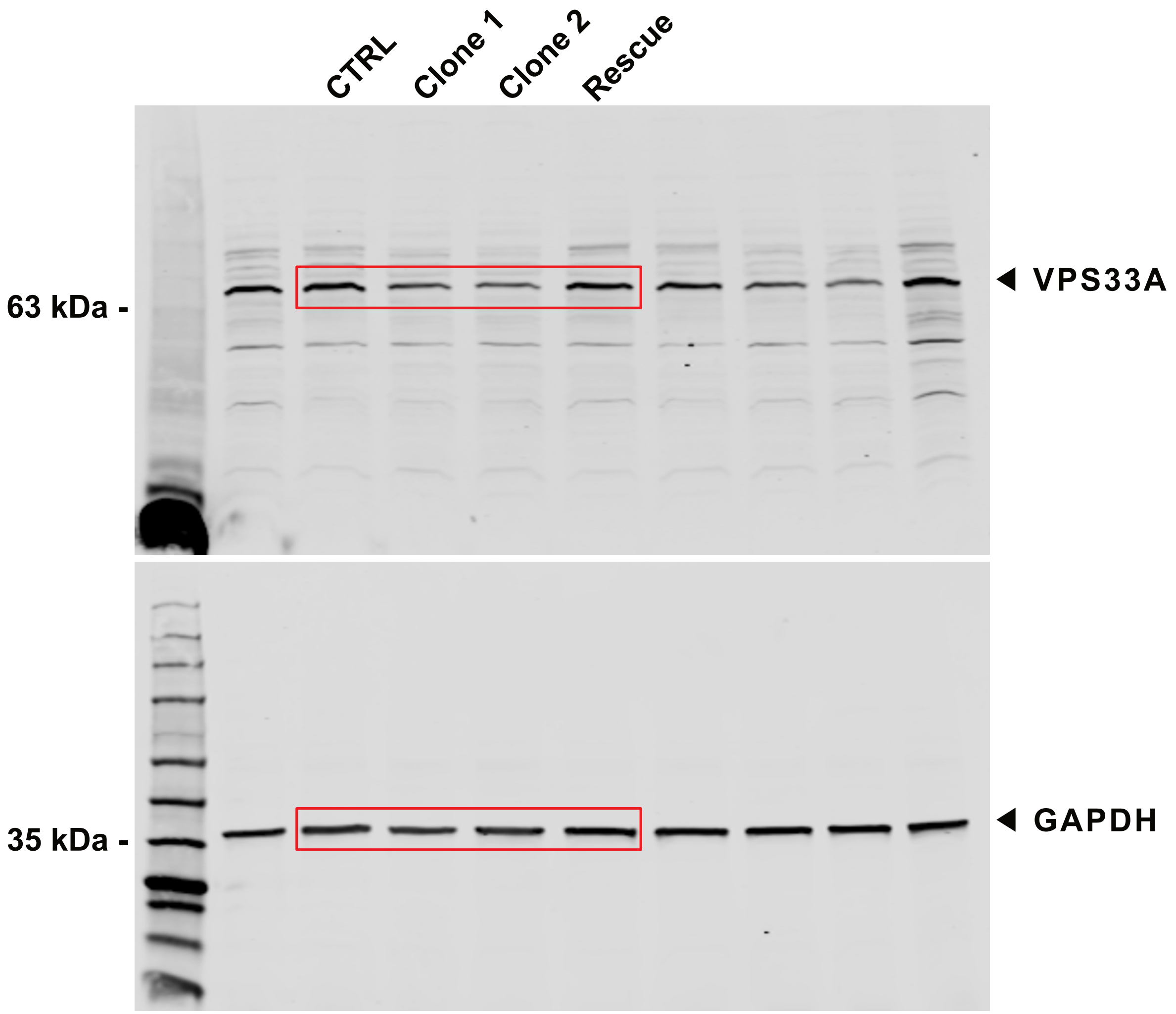


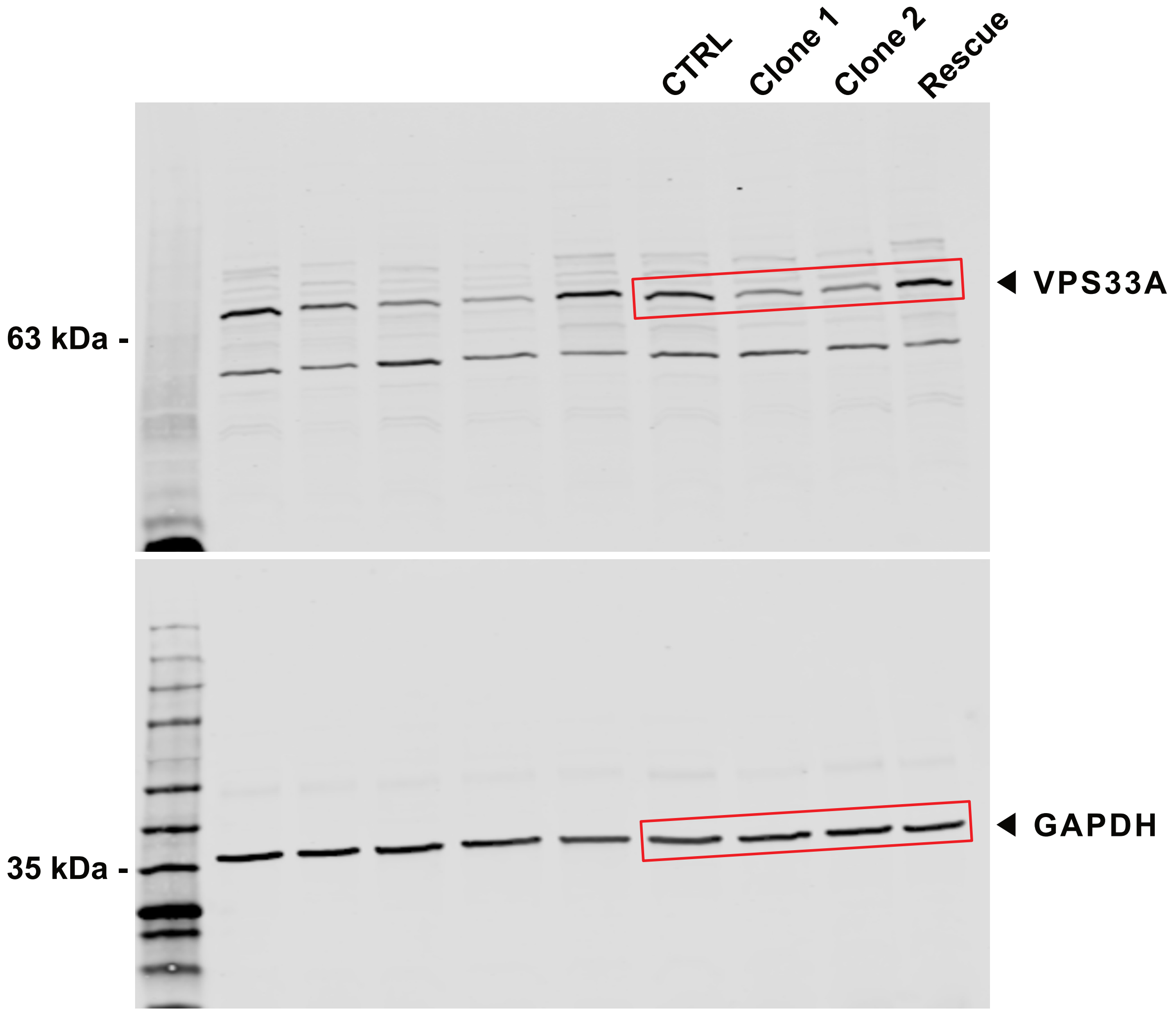

## Supplementary Figure 3E

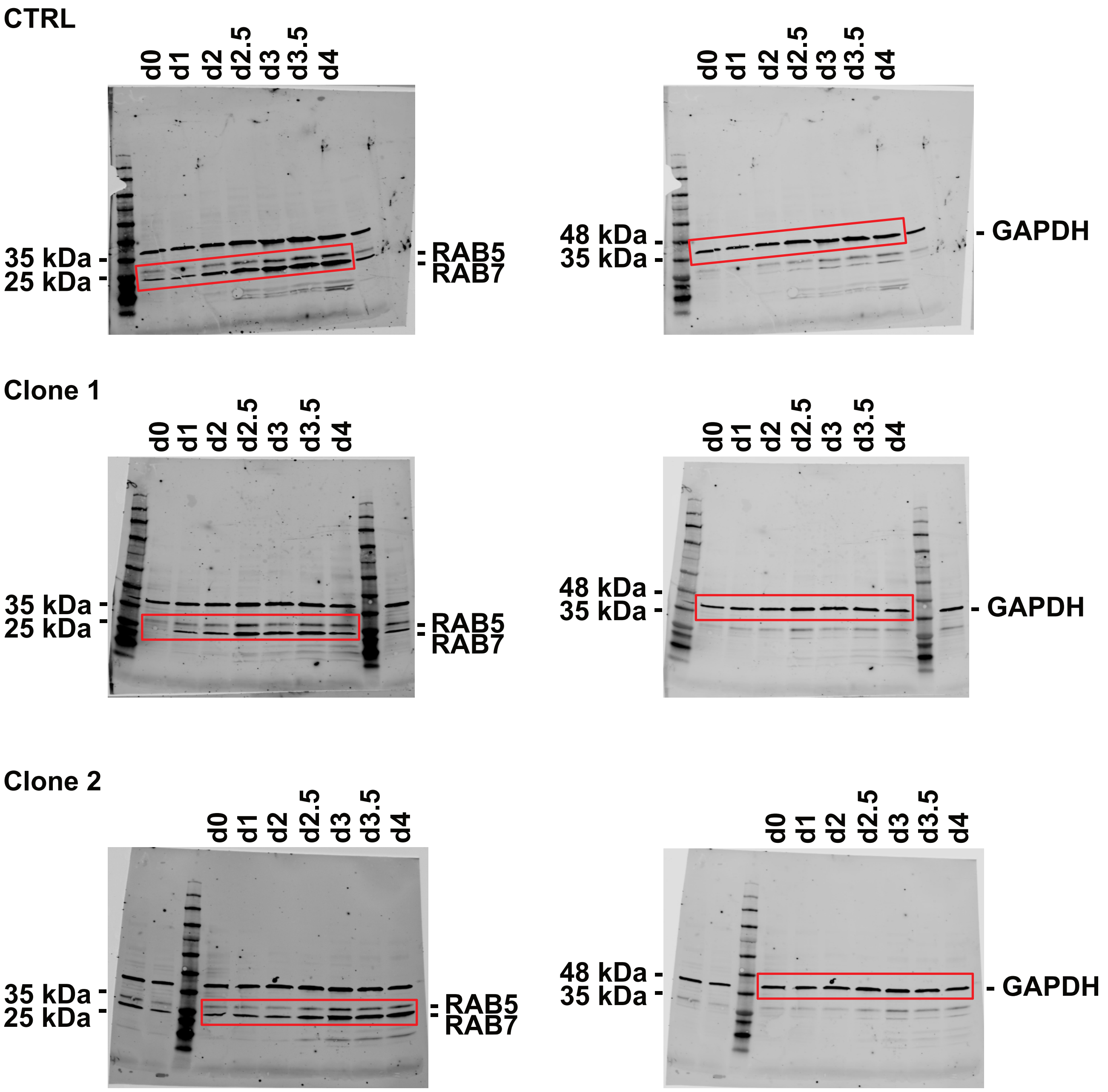


## Supplementary Figure 4B

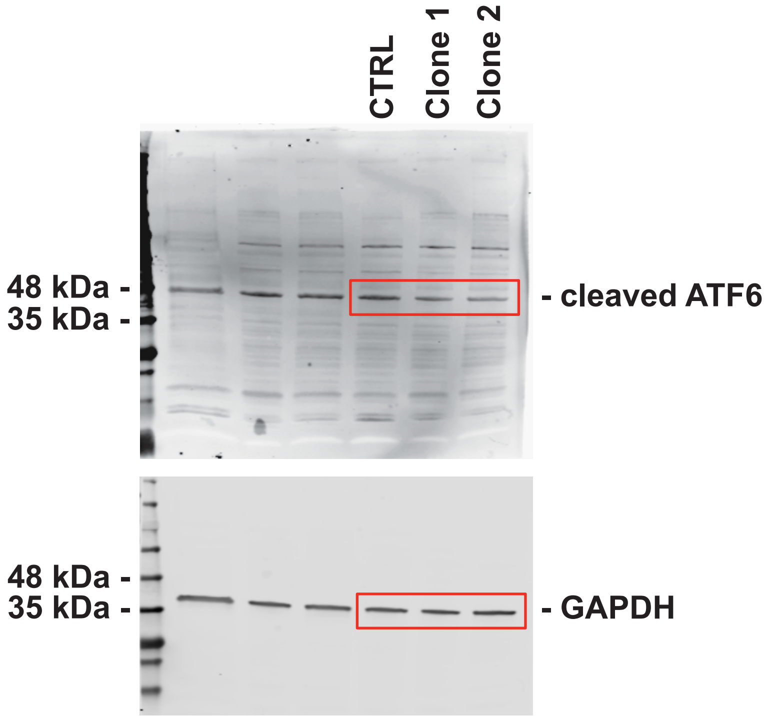

# Supplementary Figure 3F

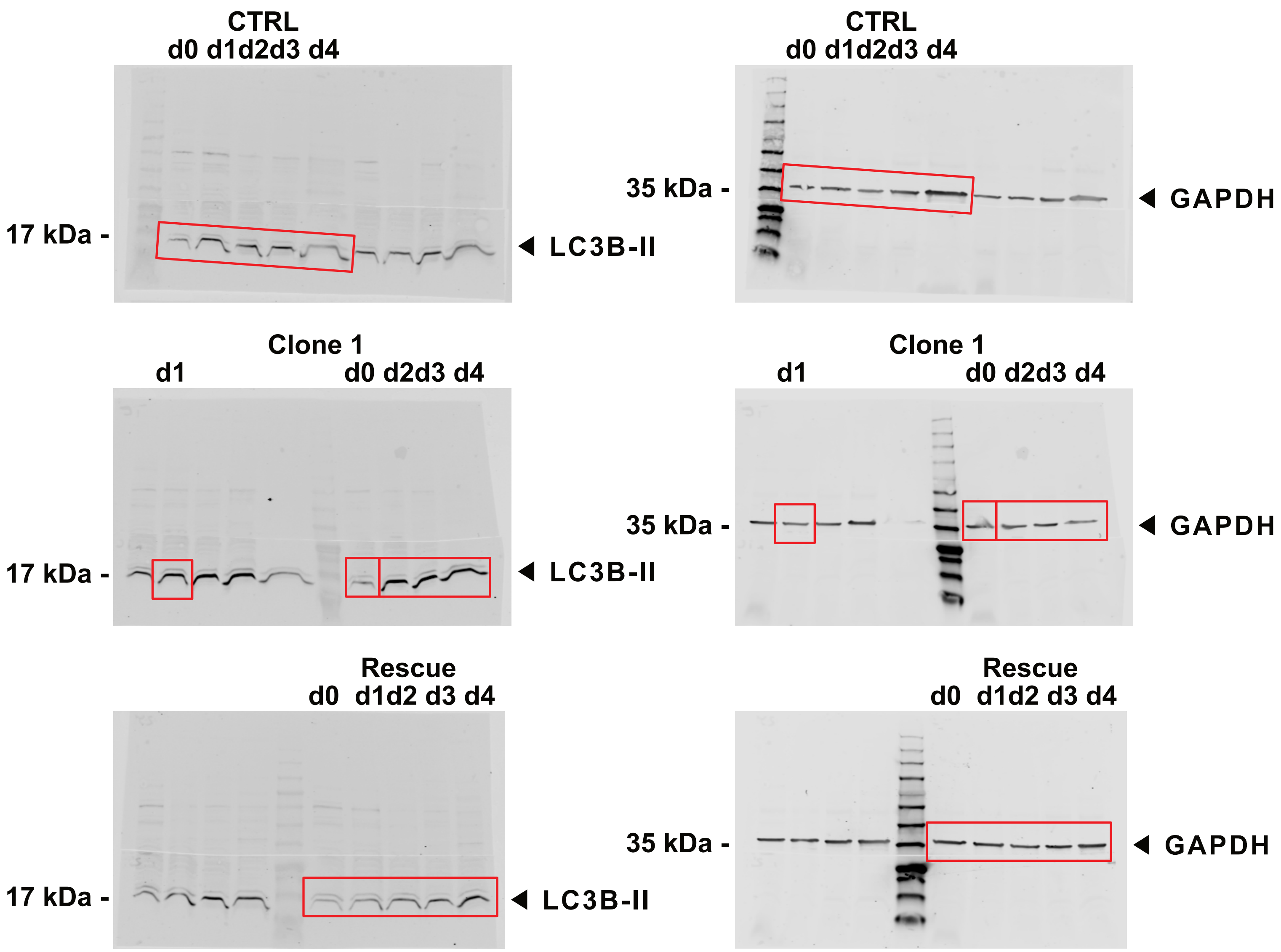

## Supplementary Figure 6B

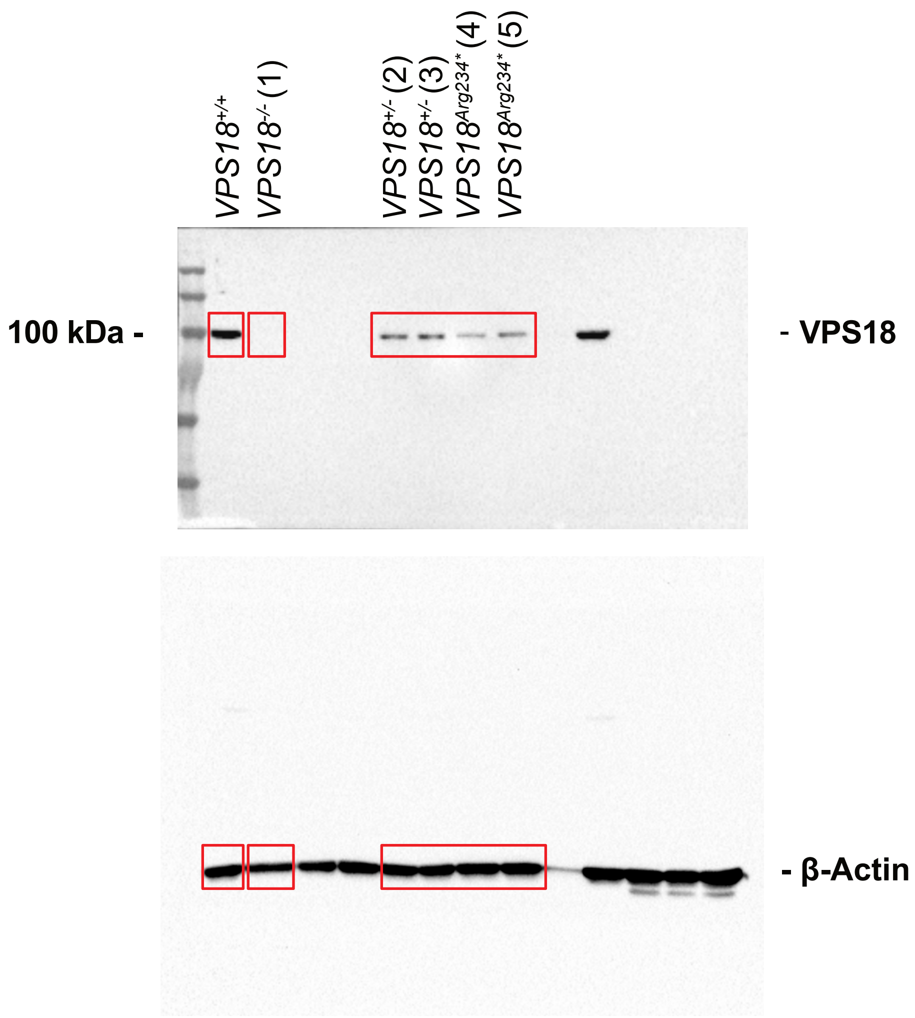


## Supplementary Figure 6C

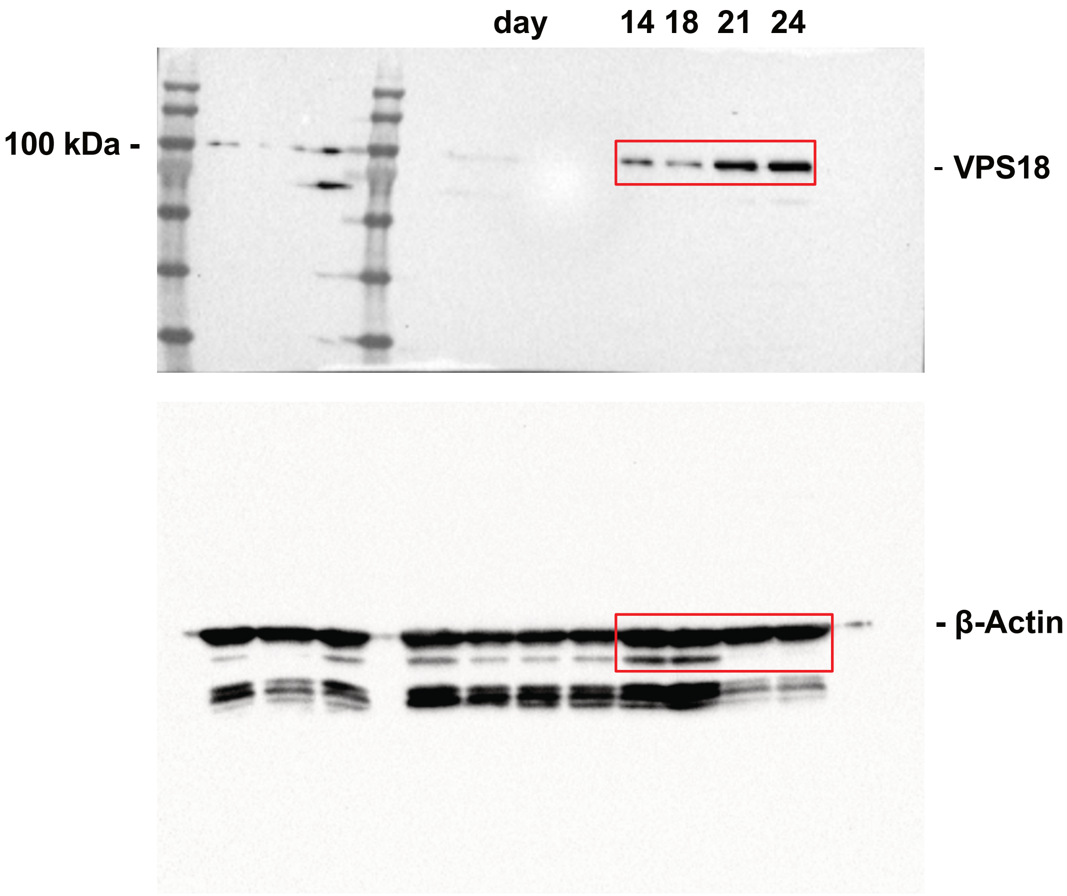

Supplement: Supplementary file 2 — Gao et al_SupplemtaryData_Original Western Blots [file 41419_2025_8338_MOESM2_ESM.pdf]
